# John Ring

**Published:** 1822-02

**Authors:** 


### JOHN RING, ESQ.

The decease of this truly excellent man, who expired of an apoplectic stroke at his house near Hanover-square, has been announced in the English and Scotch papers, with a general notice of his virtues and accomplishments. We have, however, been favoured with the following tribute to his memory, which we willingly insert.

Mr. Ring was a member of the Royal College of Surgeons in London, and of the Medical Societies of London and Paris.

He had been a pupil of the famous Percival Pott, and, by fine natural talents and an ardour to excel, attained to great eminence in his profession. He published several medical treatises, the chief of which is his work on Vaccination: he was, besides, an elegant classical scholar, and an enthusiastic lover of literature in general, which furnished him with a delightful and dignified resource for his hours of leisure. These were few; but a mind like Mr. Ring's could accomplish much in a short time. This passion for letters was the *dulce lenimen* which soothed and refreshed him after the arduous and important labours of the day. He published, at first anonymously, a poem called "The Commemoration of Handel," which he afterwards acknowledged, and reprinted two years ago, with some other pieces.

But Mr. Ring was ambitious of a higher and more durable fame, and, in January 1820, published his greatest work, a Translation of Virgil, partly original, and partly altered from Dryden and Pitt, accompanied with valuable notes. Considerable portions of the "Pastorals" and "Æneid" are his own composition; the "Georgics" he translated anew, adopting only a passage in the first, and a line or couplet occasionally from Dryden. This part of the work, in particular, is executed with a masterly hand, and with lofty and harmonious versification; combining the energy of Dryden with the mellifluous smoothness of Pope. Before the "Georgics" appeared in print, they had been honoured with the approbation of some of the first classical scholars in England.

Mr. Ring's countenance and appearance were remarkably pleasing: he was affable and easy in his manners, and fascinating in his conversation; but his feeling heart and Christian charity were what gave the truest value to his character. His benevolence, indeed, knew no bounds.

In November last, Mr. Ring was proposed, by the Bishop of St. David's, as a member of the Royal Society of Literature, and unanimously elected by the council; a signal mark of respect, and of which he was justly proud, but to which his Virgil well entitled him. His favourite amusement, even to the last, consisted in revising this great work for a second edition. He had very nearly finished his task, and may well be said to have died while completing his own honourable monument.

